# Research on provincial water resources carrying capacity and coordinated development in China based on combined weighting TOPSIS model

**DOI:** 10.1038/s41598-024-63119-3

**Published:** 2024-05-31

**Authors:** Qianying Zhu, Yi Cao

**Affiliations:** China Institute of Geo-Environment Monitoring, Beijing, 100081 China

**Keywords:** Ecology, Environmental sciences, Environmental social sciences

## Abstract

With the continuous development of the economy and society, along with the sustained population growth, the issue of water resources carrying capacity in China has attracted increasing attention. This paper constructs a model for evaluating the provincial water resources carrying capacity in China from four dimensions: water, economy, society, and ecology. Utilizing this model, we analyze the spatiotemporal variations in water resources carrying capacity among 31 provinces in China from 2005 to 2021. Additionally, we delve into the coupling coordination and influencing factors of water resources carrying capacity. The study reveals an overall increasing trend in China’s water resources carrying capacity index, with the ecological indicator exhibiting the most significant growth while the water resources sub-indicator lags behind. There are notable regional differences, with higher water resources carrying capacity observed in the eastern coastal areas and relatively lower capacity in the western regions. The ecological criterion becomes a core factor constraining water resources carrying capacity from 2005 to 2015, gradually giving way to the prominence of the social criterion since 2015. The coordination degree is relatively higher in the eastern regions, more scattered in the western regions, and relatively stable in the central regions. Based on the research findings, a series of recommendations are proposed, including strengthening environmental protection policies, optimizing water resources management mechanisms, improving water use efficiency, and promoting economic structural diversification. These suggestions aim to facilitate the sustainable development of water resources carrying capacity in China.

## Introduction

As one of the Earth’s precious natural resources, water plays a crucial role in sustaining human existence, societal development, and ecological balance^1–3^. However, with the rapid global economic growth and increasing population, the sustainable supply of water resources faces increasingly severe challenges^4,5^. China, as one of the most populous countries globally, attracts significant attention to its water resources carrying capacity (WRCC). Over the past few decades, China has achieved remarkable progress in rapid economic growth, but this achievement is accompanied by a series of contradictions, including water scarcity^6^, degradation of water ecosystems^7^, and regional imbalances in supply and demand^8^. These contradictions pose significant challenges to the utilization and governance of water resources^9,10^. The Chinese government places a high priority on environmental protection and water resources governance. In 2023, the National Development and Reform Commission and other departments issued opinions on further strengthening the conservation and intensive use of water resources, proposing to accelerate the formation of a water-saving production and lifestyle, build a water-saving society, and promote the beautiful vision of ecological civilization construction. The government emphasizes strengthening water resources management, increasing the proportion of low-consumption economy, advocating for water-saving social construction, and making ecological civilization a key focus of government work in the coming years. This study focuses on the interprovincial water resources carrying capacity and coordination in China, aiming to explore in-depth the changing trends of water resources over time and space, analyze the differences between different regions, and uncover the multidimensional factors influencing water resources carrying capacity.

Against the backdrop of global warming, accelerated urbanization, and environmental degradation, scientifically understanding the temporal and spatial characteristics of China’s WRCC, and revealing its multifaceted influences, holds significant value for formulating scientifically sound water resources management policies and promoting sustainable economic and social development. By in-depth analysis of the relationships across the water, society, economy, and ecology dimensions, this study aims to provide robust support for the sustainable utilization of China’s water resources and offer insights for other countries facing similar challenges.

The concept of water resources carrying capacity (WRCC) refers to the balanced state within a specific temporal and spatial scope between the available water resources in a region and the demands of both human and natural systems, emphasizing the sustainability and equilibrium of water resource utilization^11–13^. The research on WRCC can be traced back to the early twentieth century, evolving as understanding of water resource management and sustainable development deepened, leading to progressively more comprehensive studies.

Prior to 1950, the focus was primarily on the development and utilization of water resources to meet agricultural, industrial, and urban water needs^14^. Concerns about the carrying capacity of water resources were relatively minimal, primarily concentrated on technological and infrastructure development^15,16^. In the 1960s, as environmental issues gained attention, awareness grew regarding the potential negative impacts of water resource development on the environment. During this period, research on water resource sustainability and ecological balance emerged. As understanding of water resource sustainability deepened, the concept of integrated water resource management gradually gained prominence^17^. During this era, important studies on comprehensive planning and management of water resources emerged internationally, emphasizing the need for cooperation among different water resource utilization sectors, taking into account diverse demands^18^.

Since the twenty-first century, with the widespread dissemination of sustainable development principles and the increasing severity of climate change, research on WRCC has increasingly focused on global issues. Deeper investigations have been conducted into the impacts of climate change on water resources, the development of renewable water resources, and the regulation and governance of water resources^19,20^.

Currently, research on WRCC encompasses various aspects. Firstly, scholars such as Chen, X. J^21^, Tian, J^22^, Li, P.^23^, analyze the total quantity and availability of water resources in specific regions, including the distribution of surface water and groundwater, to assess the fundamental status of water resources. Secondly, researchers like De Mello, K.^24^, Uddin, M. G.^25^, Uddin, M. G.^26^, focus on water quality assessment, investigating the pollution status of surface water and groundwater, as well as monitoring and evaluating water quality. Thirdly, scholars such as Al-Jawad, J. Y^27^, Katusiime, J.^28^, Zhang, C. Y.^29^, utilize mathematical models, geographic information systems (GIS), remote sensing technology, and other tools to establish water resources management models and decision support systems, aiding decision-makers in scientifically planning and managing water resources. Fourthly, studies explore the relationship between socio-economic factors and water resources, as exemplified by Wang, H.^30^, Abd Ellah, R. G^31^, Viviroli, D.^32^, addressing the impact of population growth, urbanization, industrialization, and related socio-economic factors on water resource demand and proposing corresponding water resources management strategies. Fifthly, researchers like Liu, X^33^, Fang, Z.^34^, Zhang, S.^35^, investigate cross-regional water resource management issues, devising collaborative governance schemes to ensure sustainable water resource utilization. Sixthly, studies delve into ecological compensation mechanisms, as seen in the works of Gao, X^36^, Gao, X.^37^, Zheng, Q^38^, exploring how ecological compensation mechanisms can incentivize sustainable water resource utilization through economic means and encourage the protection of aquatic ecosystems. Seventhly, research related to social participation and public awareness enhancement, including Luo, P^39^, Mishra, B. K^40^, Fallon, A. L^41^, suggests that strengthening social participation and enhancing public awareness of WRCC issues can lead to more rational and sustainable water resource utilization by the public.

In summary, the research trajectory of WRCC has evolved from a singular focus on water resource development to a comprehensive consideration of sustainability and ecological balance. Throughout this process, the introduction of new theories, technologies, and methods has driven the continuous upgrading of water resource management concepts. Against this backdrop, with the development of technology, theory, and practice, WRCC evaluation has expanded from a singular focus on water quantity to a comprehensive consideration of water quality, aquatic ecology, climate, and more. Currently, WRCC assessment methods include traditional water balance methods, ecological footprint methods, water resources sustainability indices, among others. WRCC assessment places greater emphasis on the concept of sustainable development, incorporating socio-economic factors, ecological compensation mechanisms, and highlighting the importance of coordinated development and comprehensive utilization of water resources.

Overall, previous research has contributed significantly to the advancement of water resource management theory and practice. However, existing studies still have some shortcomings. Firstly, past WRCC assessment studies often focused on specific aspects such as water quantity, water quality, aquatic ecology, etc., lacking comprehensive consideration across different dimensions. Secondly, some WRCC assessment studies inadequately consider socio-economic factors. Factors like population growth, economic development, urbanization, etc., and their impacts on water resource demand are not fully considered, leading to a lack of a comprehensive understanding of human activities. Thirdly, there is a need for more in-depth research on the influencing factors of WRCC and insufficient exploration of the coordination between carrying capacity and the socio-economic environment.

The main contributions and innovations of this study are as follows: firstly, it conducts a regional division study on China’s water resources carrying capacity. By investigating the water resources carrying capacity of 31 provinces in China, the study delves into regional disparities and challenges in resource allocation. Furthermore, it employs spatial correlation analysis to identify areas of high water pressure and resource scarcity, thus expanding our understanding of the distribution pattern of China’s water resources .Secondly, it explores the coupling and coordination relationships among various subsystems within the framework of water resources carrying capacity. Through an examination of the interdependence and synergistic interactions among water resources, economy, society, and ecology subsystems, the study unveils complex relationships that exist. This research offers new perspectives for formulating comprehensive water resource management strategies.

The remaining sections of this paper are organized as follows: Sect “Research methods and data sources” includes methods and data sources; Section “Temporal and spatial variations of interprovincial WRCC in China” explores the spatial pattern of WRCC among Chinese provinces; Sect “Coupled coordination analysis of inter‑provincial WRCC in China” analyzes the coupled coordination of WRCC in China; Sect “Discussion” examines the influencing factors of WRCC differences in China, and finally, Sect “Conclusions and recommendations” presents the conclusion and discussion.

## Research methods and data sources

### Construction of evaluation model

The assessment of WRCC is a complex system influenced by various factors such as natural environment, human activities, and economic development. The vision of promoting harmonious coexistence between humans and nature outlined in China’s “14th Five-Year Plan” emphasizes the need to enhance the efficiency of natural resource utilization, including water resources. The plan calls for resource quantity management and scientific allocation, providing guidance for the interaction between water resources and economic and social activities. In 2023, the National Development and Reform Commission and other departments issued opinions on further strengthening the conservation and intensive use of water resources, emphasizing the construction of a water-saving society. Specific deployments are made in areas such as water resource management, agriculture and rural areas, industry, urban areas, and ecology, covering water resources, economy, society, and ecology. This framework further guides the research in this paper.

The construction of the indicator system in this paper is based on the research of scholars such as Yang, Z.^42^, Wang, Y.^43^, and Li, Y.^44^. Following the principles of coordinated development and comprehensive scientific selection, economic, social, ecological, and water resource-related indicators are considered together. In the selection of indicators, the actual situation of the 31 provinces in China and the operability of the indicators are comprehensively considered. The evaluation indicator system includes the goal layer, criteria layer, and indicator layer. The goal layer aims to reflect the comprehensive evaluation index of WRCC in China, fully embodying the level of WRCC in China. The WRCC system has a dual nature of natural and social attributes. The criteria layer includes four subsystems: water resources, society, economy, and ecological environment. The water resource subsystem is the carrying body, while the social, economic, and ecological environment subsystems are the carrying objects acting on the water resource system, forming a composite system through mutual interaction and promotion.

The water resource criteria layer mainly characterizes the support of water resources’ natural endowment and the level of water resource development for economic and social activities. Five indicators, such as per capita water resources and water development utilization rate, are selected to characterize this layer. The social criteria layer reflects the pressure on water resources caused by human social activities and the pursuit of high-quality life processes. Seven indicators, including per capita GDP and urbanization rate, are selected to represent this layer. The economic criteria layer reflects the impact of economic activities on WRCC, and five indicators, including water consumption per unit of GDP, are selected to characterize this layer. The ecological environment criteria layer reflects the support of the ecological environment system for water resources through environmental pollution and the effectiveness of environmental governance. Eight indicators, including ecological water consumption and sewage treatment rate, are selected to represent this layer. The selected 25 evaluation indicators have low correlation, avoiding redundancy. The evaluation indicator system is shown in Table 1.

**Table 1:** Evaluation index system for China’s WRCC.

Objective layer	Criteria layer	Indicator layer	Symbol	Unit	Indicator property
WRCC	Water Resources	Per capita water resources	X1	m^3^/ people	+
		Precipitation	X2	10^8^m^3^	+
		Surface water supply	X3	10^8^m^3^	+
		Groundwater supply	X4	10^8^m^3^	+
		Water development utilization rate	X5	%	−
	Society	Per capita GDP	X6	People /km^2^	+
		Urbanization rate	X7	%	+
		Proportion of the three industries	X8	%	+
		Water consumption per unit of arable land	X9	10^8^m^3^	−
		Per capita domestic water consumption	X10	L	−
		Water supply coverage	X11	%	+
		Agricultural irrigation area	X12	10^3^hm	−
	Economy	Economic density	X13	Yuan/km^2^	+
		Gross domestic product index	X14	%	+
		Industrial water consumption	X15	10^8^m^3^	−
		Water consumption per 10,000 yuan of GDP	X16	m^3^/yuan	−
		Water consumption per 10,000 yuan of industrial added value	X17	m^3^/10^4^yuan	−
	Ecology	Ecological water consumption	X18	10^8^m^3^	+
		Greening coverage in built-up areas	X19	%	+
		Discharge of industrial wastewater	X20	10^8^m^3^	−
		Total emission of pollutants	X21	Ten thousand tons	−
		Forest coverage	X22	%	+
		Industrial wastewater treatment capacity	X23	Tons /day	+
		Per capita ecological environment water consumption	X24	m^3^/ people	+
		Sewage treatment rate	X25	%	+

### Research methods

#### Calculation of WRCC index

Entropy weight method is an objective weighting approach that can effectively analyze the contribution of each evaluation indicator to the WRCC based on objective weights^45^. Technique for order preference by similarity to ideal solution (TOPSIS) is an efficient decision analysis method based on multiple indicators and objectives. It calculates the Euclidean distance between the evaluation object and the best and worst options, providing the proximity of each evaluation object to the optimal solution. The final ranking is determined based on this proximity, offering a comprehensive analysis of each evaluation object. The construction of the entropy weight TOPSIS model is outlined below.

- (1) Formation of decision matrix. For m evaluated provinces and n evaluation indicators, where ${x}_{ij}$ represents the value of the j-th indicator in the i-th province, the initial matrix X is represented as follows:
  1 $$X=\left[\begin{array}{ccc}{x}_{11}& \cdots & {x}_{1n}\\ \vdots & \ddots & \vdots \\ {x}_{m1}& \cdots & {x}_{mn}\end{array}\right]$$
- (2) Normalization of decision matrix. The judgment matrix is normalized to obtain the normalized judgment matrix Y:
  2 $$y_{ij} = \frac{{x_{ij} - \min (x_{ij} )}}{{\max (x_{ij} ) - \min (x_{ij} )}}\,\left( {\text{for positive indicators}} \right)$$
  3 $$y_{ij} = \frac{{\max (x_{ij} ) - x_{ij} }}{{\max (x_{ij} ) - \min (x_{ij} )}}\,\left( {\text{for negative indicators}} \right)$$
- (3) Determination of composite weights for indicators.
  ① Calculation of entropy weight using the entropy method, denoted as $\omega_{j}^{\prime }$, is computed for the jth indicator with the following formula:
  4 $$\omega_{j}^{\prime } = \frac{{1 - e_{j} }}{{\mathop \sum \nolimits_{j = 1}^{n} \left( {1 - e_{j} } \right)}}$$
  where, information entropy ${\text{e}}_{i}$, normalized matrix ${f}_{ij}$, are calculated using the following formulas:
  5 $${\text{e}}_{\text{j}}=-\frac{1}{\text{lnm}}\sum_{\text{i}=1}^{\text{m}}\left[{\text{f}}_{\text{ij}}\times {\text{lnf}}_{\text{ij}}\right]$$
  6 $${\text{f}}_{{{\text{ij}}}} { = }\frac{{{\text{y}}_{{{\text{ij}}}} }}{{\sum\nolimits_{{\text{j = 1}}}^{{\text{m}}} {{\text{y}}_{{{\text{ij}}}} } }}$$
  ②Coefficient of variation method to calculate weight, the formula is as follows:
  7 $$C = \frac{1}{{R_{0} }}\sqrt {\frac{1}{m}\mathop \sum \limits_{i = 1}^{m} \left( {R_{I} - R_{0} } \right)^{2} }$$
  8 $$\omega_{j}^{\prime \prime } = \frac{C}{{\mathop \sum \nolimits_{j = 1}^{n} C}}$$
  where C is the coefficient of variation, ${R}_{0}$ is the average value of the jth indicator, ${R}_{i}$ is the original value of the jth indicator, and $\omega_{j}^{\prime }$ is the weight of the jth indicator.
  ③AHP method weights. AHP is a practical multi-criteria decision-making method that involves pairwise comparisons of various indicators to construct a judgment matrix. The method uses the eigenvalue method to determine the weights of each indicator. In this study, a 9-point scale was used to quantify the importance of each indicator, following the calculation steps and formulas from Yu, D^46^ and other studies. Consistency indicators and average random consistency indicators were calculated for the analysis results, and the consistency test results were used for combined weight analysis. $\omega_{j}^{\prime \prime \prime }$ represents the AHP weight. The combined weight ${\omega }_{j}$ results are shown in Table 2, and the calculation formula is as follows:
  9 $$\omega_{j} = \frac{{\omega_{j}^{\prime } + \omega_{j}^{\prime \prime } + \omega_{j}^{\prime \prime \prime } }}{3}$$
- (4) Calculation of WRCC index.
  Calculate the distance between the WRCC index and the positive and negative ideal values using the Euclidean distance. D^+^ represents the distance between the i-th index and ${Z}^{+}$, D^-^ represents the distance between the i-th index and ${Z}^{-}$, and D represents the WRCC index for China. A larger value indicates a higher level of safety. The calculation formula is as follows:
  10 $$D_{i}^{ + } = \sqrt {\sum\limits_{j = 1}^{n} {(Z_{i}^{ + } - y_{ij} )^{2} } }$$
  11 $$D_{i}^{ - } = \sqrt {\sum\limits_{j = 1}^{n} {(Z_{i}^{ - } - y_{ij} )^{2} } }$$
  12 $$\text{D}=\frac{{D}_{j}^{-}}{{D}_{j}^{+}{+D}_{j}^{-}}$$
  where: ${Z}^{+}$ is the positive ideal solution, ${Z}^{-}$ is the negative ideal solution, and $Z^{ + } = \max \left\{ {y_{ij} } \right\}$ and $Z^{ - } = \min \left\{ {y_{ij} } \right\}$.
  Coupling Coordination Analysis.
  The coupling coordination model analyzes the level of coordinated development between two or more systems, consisting of coupling degree (C) and comprehensive development index (T). The calculation formula is as follows:
  13 $$C={\left[\frac{{Y}_{1}\times {Y}_{2}\times {Y}_{3}\times {Y}_{4}}{{\left(\frac{{Y}_{1}+{Y}_{2}+{Y}_{3}+{Y}_{4}}{4}\right)}^{4}}\right]}^\frac{1}{4}$$
  14 $$T=\alpha {Y}_{1}+{\beta Y}_{2}+{\gamma Y}_{3}+\delta {Y}_{4}$$
  15 $$D=\sqrt{C\times T}$$
  where: ${Y}_{1}$, ${Y}_{2}$, ${Y}_{3}$, ${Y}_{4}$ are the evaluation indices corresponding to the water resources, economic, social, and ecological criteria layers, and these indices are obtained by synthesizing the normalized values of various indicators within the four criteria layers with their combination weights; C represents coupling degree, T represents comprehensive development index; α, β, γ, δ are undetermined coefficients with the condition α + β + γ + δ = 1, considering equal importance of mutual constraints among the four systems, set α = β = γ = δ = 0.25; D is the coupling coordination degree, reflecting the level of coordinated development among the systems and the overall development level of the region, and the magnitude of D is directly proportional to the level of development.

**Table 2:** Integrated weight calculation results for each indicator.

Index codes	Weights calculated by different methods			Composite weight
	$\omega_{j}^{\prime }$	$\omega_{j}^{\prime \prime }$	$\omega_{j}^{\prime \prime \prime }$	
X1	0.196	0.249	0.178	0.178
X2	0.031	0.024	0.028	0.028
X3	0.047	0.048	0.047	0.047
X4	0.067	0.088	0.077	0.077
X5	0.009	0.002	0.016	0.016
X6	0.040	0.033	0.058	0.058
X7	0.023	0.013	0.018	0.018
X8	0.029	0.018	0.024	0.024
X9	0.014	0.005	0.020	0.020
X10	0.021	0.013	0.017	0.017
X11	0.009	0.002	0.007	0.007
X12	0.020	0.011	0.016	0.016
X13	0.149	0.198	0.152	0.152
X14	0.019	0.008	0.013	0.013
X15	0.011	0.004	0.008	0.008
X16	0.007	0.001	0.006	0.006
X17	0.012	0.004	0.037	0.037
X18	0.069	0.074	0.064	0.064
X19	0.015	0.006	0.011	0.011
X20	0.009	0.002	0.006	0.006
X21	0.013	0.005	0.016	0.016
X22	0.033	0.030	0.031	0.031
X23	0.049	0.047	0.048	0.048
X24	0.082	0.089	0.075	0.075
X25	0.028	0.025	0.027	0.027

#### Geodetector

The geodetector is a statistical analysis method for detecting the spatial differentiation of variables and their influencing factors. This method has many advantages, including a small sample size requirement (less than 30 samples), immunity to collinearity, and the ability to investigate the interaction of variables47,48,49[47-49]. It is currently widely used in various disciplines such as geography, economics, and sociology. The formula for calculating the q value of the Geodetector is as follows:

16 $$q=1-\frac{1}{N{\sigma }^{2}}\sum_{h=1}^{L}Nh{\sigma }_{h}^{2}$$

where: q is the detection value of the impact factor on the spatial heterogeneity of China’s WRCC, q ∈ [0,1], a larger q value indicates a stronger explanatory power of the factor for the spatial heterogeneity of the dependent variable. L is the stratification of the influencing factors; h is the sample size of the driving factor in the study area, and σ^2^ is the variance of the dependent variable in the study area.

### Data sources

This study focuses on 31 provinces in China (excluding Hong Kong, Macau, and Taiwan due to some data unavailability) as the research area. Economic indicators, population statistics, water consumption, precipitation, and water supply data were sourced from the China statistical yearbook. Ecological water consumption and sewage discharge data were obtained from the China environmental statistical yearbook. Additional data were sourced from individual provincial statistical yearbooks, with any missing data supplemented using information published by the respective provincial statistical authorities.

## Temporal and spatial variations of interprovincial WRCC in China

### Temporal series analysis of interprovincial WRCC in China

**Figure 1:**
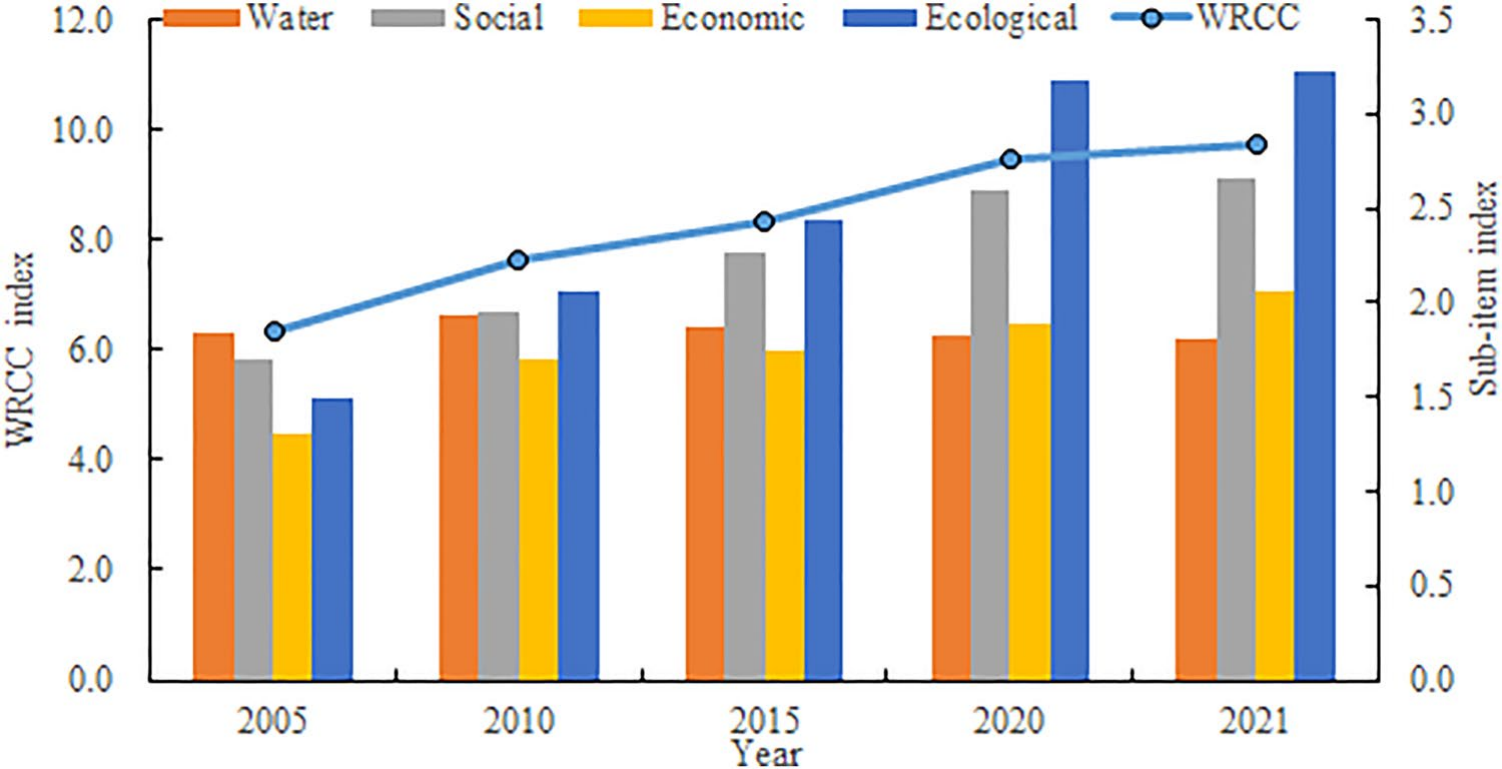
Evolution trend of China’s WRCC index from 2005 to 2021.

The evaluation model for interprovincial WRCC in China is constructed with 25 assessment indicators across four dimensions: water resources, society, economy, and ecology. The weights of each indicator are determined using entropy weight method, analytic hierarchy process (AHP), and coefficient of variation method. Subsequently, the TOPSIS method is employed to calculate the WRCC indices for 31 Chinese provinces from 2005 to 2021 (Fig. 1). The WRCC index has exhibited a continuous increase since 2005, rising from 6.357 in 2005 to 9.749 in 2021, marking a substantial growth of 53.4%. However, the magnitudes of change in the four sub-indicators of the WRCC index vary. The ecological indicator has shown the most significant growth, increasing by 115.2% since 2005, indicating a considerable emphasis on environmental protection and increased resource investment by the Chinese government. Meanwhile, the economic and social sub-indicators have increased by 57.0% and 55.6%, respectively, since 2005, reflecting notable achievements in economic development with simultaneous attention to social development. However, the water resources sub-indicator has remained stagnant, with a 1.6% decrease since 2005, primarily due to the overall stability of per capita water resources in China, coupled with reduced potential for water resources development and utilization resulting from water pollution and irrational usage.

Table 3 presents the WRCC index values of 31 provinces in China from 2005 to 2021. It can be observed that the spatial distribution of China's WRCC index is positively correlated with the inter-provincial economic level. On the one hand, in 2021, economically developed regions in the eastern part of China, such as Jiangsu, Beijing, inner Mongolia, Shanghai, Henan, and Guangdong, have higher WRCC indices than other provinces. In contrast, economically underdeveloped provinces in the western part, such as Gansu, Guizhou, Qinghai, and Ningxia, generally exhibit lower WRCC indices. On the other hand, considering the growth rate of the WRCC index since 2005, economically developed provinces in the east generally outperform other economically underdeveloped regions. For instance, the WRCC index growth rates since 2005 for provinces like Jiangsu, Beijing, Shanghai, Hubei, Guangdong, and Tianjin are 0.236, 0.169, 0.163, 0.147, 0.127, and 0.127, respectively. In contrast, provinces like Yunnan, Hainan, Heilongjiang, Xinjiang, and Tibet have WRCC index growth rates all less than 0.1, ranking lower.

**Table 3:** WRCC indices of Chinese provinces from 2005 to 2021.

Serial number	Province	Water resources carrying capacity index				
		2005	2010	2015	2020	2021
1	Beijing	0.233	0.285	0.336	0.389	0.401
2	Tianjing	0.200	0.231	0.264	0.294	0.327
3	Hebei	0.239	0.276	0.285	0.332	0.351
4	Shanxi	0.185	0.226	0.234	0.257	0.270
5	Inner Mongolia	0.223	0.260	0.309	0.357	0.370
6	Liaoning	0.215	0.252	0.274	0.296	0.304
7	Jilin	0.193	0.225	0.256	0.295	0.288
8	Heilongjiang	0.228	0.260	0.302	0.303	0.304
9	Shanghai	0.202	0.260	0.305	0.348	0.365
10	Jiangsu	0.232	0.306	0.369	0.441	0.468
11	Zhejiang	0.248	0.277	0.290	0.318	0.330
12	Anhui	0.174	0.232	0.260	0.290	0.296
13	Fujian	0.210	0.254	0.276	0.308	0.332
14	Jiangxi	0.191	0.252	0.262	0.283	0.296
15	Shandong	0.239	0.262	0.283	0.328	0.337
16	Henan	0.226	0.267	0.283	0.351	0.353
17	Hubei	0.183	0.224	0.248	0.295	0.331
18	Hunan	0.187	0.246	0.268	0.293	0.309
19	Guangdong	0.226	0.293	0.312	0.343	0.353
20	Guangxi	0.175	0.233	0.261	0.282	0.291
21	Hainan	0.190	0.224	0.230	0.258	0.268
22	Chongqing	0.184	0.222	0.250	0.273	0.279
23	Sichuan	0.183	0.219	0.246	0.277	0.286
24	Guizhou	0.156	0.200	0.233	0.258	0.262
25	Yunnan	0.194	0.221	0.245	0.269	0.275
26	Tibet	0.297	0.310	0.294	0.325	0.323
27	Shaanxi	0.200	0.229	0.252	0.274	0.287
28	Gansu	0.166	0.189	0.217	0.259	0.266
29	Qinghai	0.142	0.189	0.201	0.237	0.245
30	Ningxia	0.139	0.185	0.203	0.234	0.236
31	Xinjiang	0.297	0.326	0.280	0.414	0.343

Since 2005, the WRCC indices of all 31 provinces have increased, indicating significant achievements in China’s water resources and ecological protection. However, the magnitude of the increase varies. Jiangsu province stands out with a WRCC index growth exceeding 100%, significantly higher than other provinces. Eighteen provinces, including Shanghai and Hubei, have WRCC index growth rates exceeding 50%, ranking at the forefront. In contrast, four provinces, including Zhejiang, Heilongjiang, Xinjiang, and Tibet, have WRCC index growth rates below 30%, indicating relatively lower increases.

### Spatial variation analysis of provincial WRCC in China

This section will further analyze the regional differences in provincial WRCC indices in China. Firstly, based on the WRCC indices of 31 provinces from 2005 to 2021, the standard score method is employed to categorize them into four levels: I, II, III, and IV, as indicated in Table 4.

**Table 4:** Classification criteria for WRCC levels.

Grading standards	Level I	Level II	Level III	Level IV
Division basis	(0, V-B]	(V-B, V]	(V, V + B]	(V + B, 1]
	(0, 0.048]	(0.048, 0.079]	(0.079, 0.109]	(0.109, 0.199]

**Figure 2:**
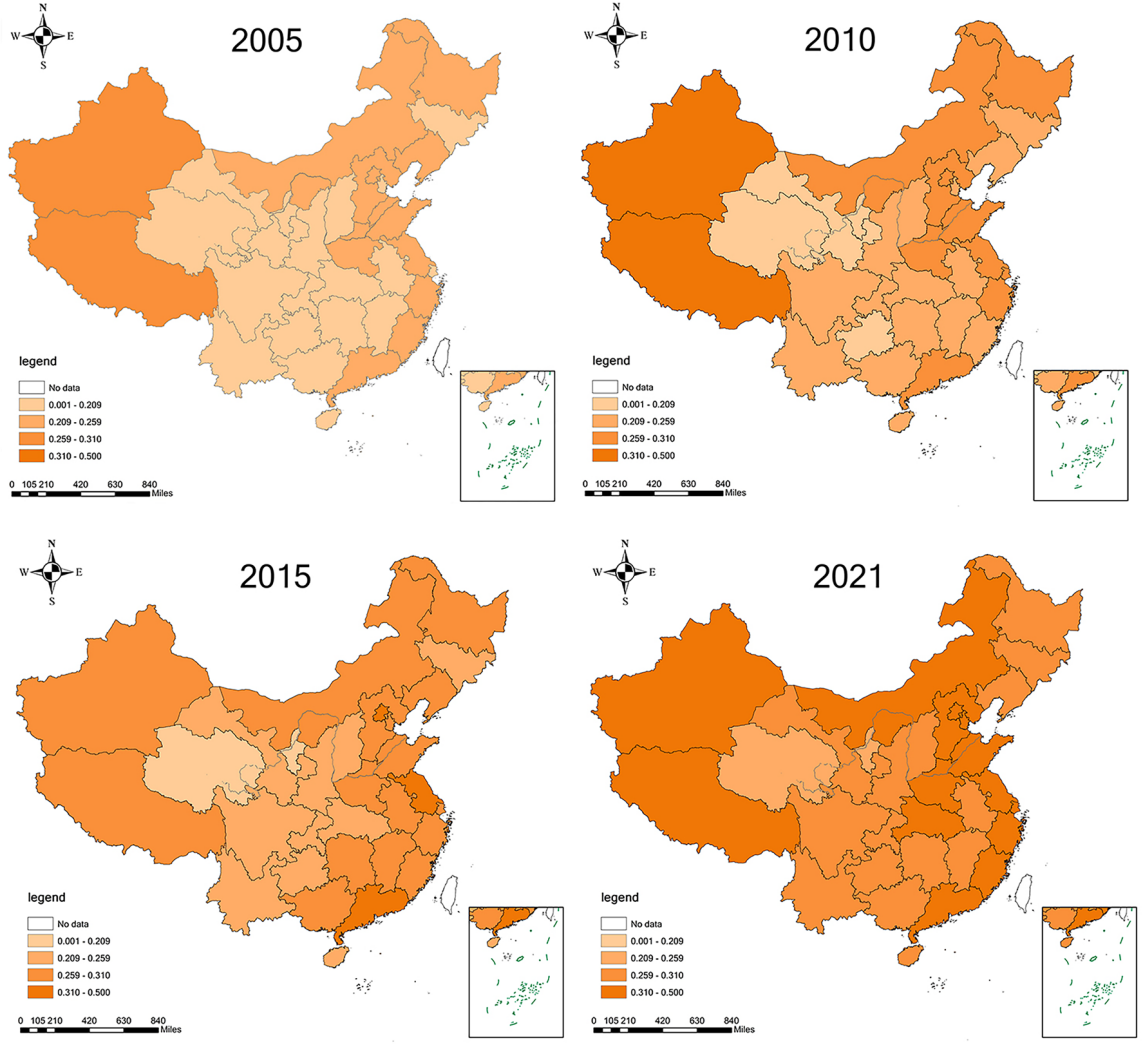
Spatial pattern of WRCC indices in China from 2005 to 2021. This figure was created for this study. The base map is based on the 2019 China map—survey number GS(2019)1822, and the drawing software used is ArcGIS 10.7.

Subsequently, according to the level classification principles in Table 4, we classify the WRCC indices calculated for the years 2005, 2010, 2015, and 2021 into four levels. The results are presented in Fig. 2, where darker colors indicate higher WRCC indices, reflecting stronger water resource carrying capacity. As depicted in Fig. 2, in 2005, there were 18, 11, 2, and 0 provinces in levels I, II, III, and IV, respectively, among the 31 Chinese provinces. In 2010, these numbers changed to 7, 35, 13, and 9 provinces for levels I, II, III, and IV, respectively. By 2021, there were 0, 2, 15, and 14 provinces in levels I, II, III, and IV, respectively. It is evident that since 2005, the provinces in level I have significantly decreased, while those in level IV have increased, primarily in North China, Central China, and South China. Specifically, coastal provinces in the east boast relatively abundant water resources, high economic efficiency of water resources, and substantial pollution control efforts. These regions consistently maintain a high level of WRCC due to their favorable geographical location, developed economic foundations, and advanced governance experience.

The three provinces in the northeast are vital industrial and grain bases in China, with a good economic foundation and abundant natural resources providing a solid basis for WRCC in the region. However, since 2015, their ranking in the country has declined, mainly due to the lack of significant upgrades in the economic industry structure, with a large proportion of high-energy and high-water-consuming industries. For example, in 2021, the water consumption per unit value added of industrial enterprises in the three northeastern provinces was 30.1 cubic meters, higher than the national average of 28.8 cubic meters, 2.3 times that of Zhejiang, and 5.9 times that of Beijing.

Western provinces generally exhibit a lower level of WRCC, primarily due to the relatively harsh natural environment in the western region and lower economic development levels. For instance, Gansu’s per capita GDP is 41,000 yuan, 51.4% of the national average and 29.8% of Jiangsu. Overall, despite China’s significant improvement in WRCC amid rapid economic development, regional imbalances remain prominent.

**Figure 3:**
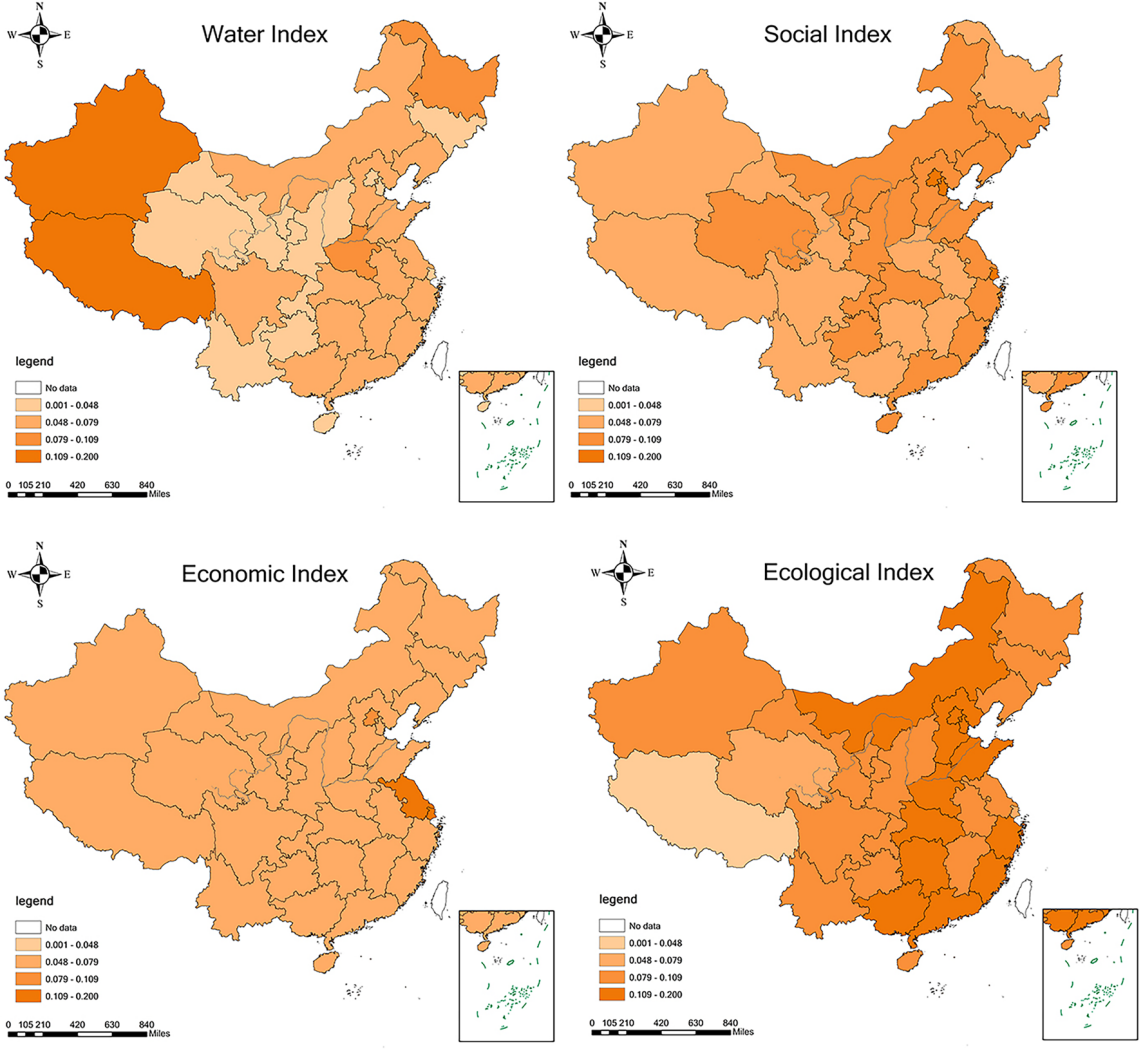
Spatial pattern of criteria-level WRCC indices in China for the year 2021. This figure was created for this study. The base map is based on the 2019 China map—survey number GS(2019)1822, and the drawing software used is ArcGIS 10.7.

Figure 3 illustrates the spatial pattern of the four sub-indicators of WRCC across 31 provinces in China in 2021. Darker colors indicate a stronger supporting role of the respective sub-indicator for sustainable water resource development, contributing to higher WRCC. The four sub-indicators are as follows:

Water resources indicator: represents the natural environment's capacity to support WRCC. Regions with more water resources and lower utilization rates exhibit greater capacity to support economic and population growth. Notably, provinces in the east and south, such as Zhejiang, Jiangsu, and Hubei, demonstrate significantly higher per capita water resources compared to the national average, providing robust support for regional economic and social development. In contrast, some western provinces, excluding Xinjiang and Tibet, face relative scarcity due to high water resource development, limiting local WRCC.

Social indicator: represents the supporting capacity of the national social development level for WRCC. Higher social development levels under similar water resource conditions contribute to greater WRCC. The social indicator in 2021 exhibits significant regional disparities, with economically developed regions like Beijing and Shanghai maintaining relatively high levels. This reflects the dominant influence of economic factors, where per capita GDP has a substantial impact on the overall index. Regional disparities also highlight differences in water resource utilization efficiency and the urban-rural gap.

Economic indicator: represents the supporting capacity of the economic development level for WRCC. It is closely related to economic levels, industrial water consumption, water use per unit of GDP, and water use per unit of industrial value-added. The economic indicators of provinces and autonomous regions in China show diverse characteristics. Eastern coastal areas like Beijing, Shanghai, and Jiangsu consistently maintain high economic levels, driven by early open-door policies and advanced industrial structures. Western regions, including Chongqing and Shaanxi, demonstrate steady economic growth, indicating achievements in narrowing the economic gap with eastern regions.

Ecological indicator: represents the supporting capacity of ecological protection for WRCC. It is closely related to ecological water consumption, urban green coverage, emissions of waste and sewage, total pollutant emissions, forest coverage, industrial wastewater treatment capacity, per capita ecological environment water consumption, and sewage treatment rate. The ecological indicators reveal the performance of provinces, municipalities, and autonomous regions in environmental protection and sustainable development. Developed eastern regions, such as Beijing, Shanghai, and Zhejiang, show gradual improvement in ecological indicators, indicating proactive measures in environmental governance and sustainable development.

## Coupled coordination analysis of inter-provincial WRCC in China

In the context of the overall coupling coordination and the coupling coordination between the criteria layer of water resources, economy, society, and ecology, the coupling degree and comprehensive development index are introduced for coupled coordination analysis. The results are presented in Table 5. Overall, the coordination degree in the eastern region is relatively high. Provinces in the eastern region, such as Beijing, Shanghai, Jiangsu, and Zhejiang, demonstrated a generally high level of coordination from 2005 to 2021, reflecting the achievements in the coordinated development of their economy and environment. The coordination degree in the western region is more dispersed. Provinces in the western region, such as Xinjiang, Qinghai, and Ningxia, showed relatively low and fluctuating coordination degrees throughout the period, with Xinjiang experiencing some improvement after 2016 but still maintaining a lower overall level. The central region exhibits a relatively stable overall coordination degree. Provinces in the central region, including Henan, Hubei, and Hunan, demonstrated relatively stable coordination degrees throughout the entire period, indicating positive efforts in industrial restructuring and environmental protection in the central region.

**Table 5:** Coupling coordination index of 31 provinces in China.

Serial number	Province	Coupling degree	Comprehensive development index	Coupling coordination index
		C-value	T-value	D-value
1	Beijing	0.845	0.100	0.291
2	Tianjing	0.855	0.082	0.264
3	Hebei	0.930	0.088	0.286
4	Shanxi	0.954	0.068	0.254
5	Inner Mongolia	0.935	0.093	0.294
6	Liaoning	0.956	0.076	0.269
7	Jilin	0.952	0.072	0.262
8	Heilongjiang	0.972	0.076	0.272
9	Shanghai	0.904	0.091	0.287
10	Jiangsu	0.930	0.117	0.330
11	Zhejiang	0.946	0.082	0.279
12	Anhui	0.978	0.074	0.269
13	Fujian	0.931	0.083	0.278
14	Jiangxi	0.976	0.074	0.269
15	Shandong	0.968	0.084	0.286
16	Henan	0.948	0.088	0.289
17	Hubei	0.950	0.083	0.280
18	Hunan	0.964	0.077	0.273
19	Guangdong	0.956	0.088	0.291
20	Guangxi	0.962	0.073	0.265
21	Hainan	0.960	0.067	0.254
22	Chongqing	0.944	0.070	0.257
23	Sichuan	0.972	0.072	0.264
24	Guizhou	0.967	0.066	0.252
25	Yunnan	0.961	0.069	0.257
26	Tibet	0.876	0.081	0.266
27	Shaanxi	0.955	0.072	0.262
28	Gansu	0.952	0.067	0.252
29	Qinghai	0.967	0.061	0.244
30	Ningxia	0.813	0.059	0.219
31	Xinjiang	0.951	0.086	0.286

The temporal changes in the coordination of water resources, economy, society, and ecology in various provinces of China reveal the interrelationships among multidimensional indicators. Firstly, there is a significant regional disparity, with the coordination degree showing notable differences among provinces. The eastern coastal regions exhibit relatively higher coordination, while the western and northern regions demonstrate comparatively lower coordination. This reflects the uneven distribution of regional water resources, the heterogeneity of economic structures, and varying levels of social development. Secondly, ecological coordination tends to be low, with most provinces showing relatively low ecological coordination. This indicates a disconnect between the ecological environment and socio-economic development, possibly due to overexploitation and environmental pollution, exerting pressure on the ecosystems. Thirdly, there is a correlation between water resources and socio-economic factors. Some developed provinces exhibit higher water resources-economic coordination, reflecting successful mutual promotion between water resources and socio-economic development achieved through technological innovation and coordinated water resource management for sustainable development. Fourthly, population factors play a significant role, with regions of high population density generally having lower coordination. This suggests that population factors play an important role in the coordination of water resources, economy, society, and ecology, and excessive population may lead to over-demand for water resources, affecting overall coordination. Lastly, economic structural diversity contributes to more effective synergy between water resources and economic development. In contrast, provinces relying on a single industry may be more susceptible to resource pressure.

Analysis of the reasons for the differences in water resources coupling coordination among Chinese provinces: On one hand, regional differences primarily stem from variations in natural conditions and local policies, requiring differentiated water resource management policies and regional coordinated development to enhance coordination. On the other hand, low ecological coordination may result from factors such as overexploitation and inadequate environmental protection measures, necessitating strengthened policies for ecological protection and sustainable utilization. Furthermore, the association between water resources and socio-economics needs to be achieved by improving water resource utilization efficiency and fostering technological innovation. Population factors require regulation through population policies and urban planning to achieve balanced development among population, resources, and the environment. Additionally, promoting economic structural diversity helps enhance adaptability and resilience, necessitating encouragement for diversified industrial development.

### Analysis of influencing factors on provincial WRCC in China

In light of the significant differences in WRCC levels identified in the previous sections, mainly attributed to variations in relevant indicators, this section employs geodetectors to quantitatively identify the influence of spatial differentiation on each detecting factor (Q-statistic).

**Figure 4:**
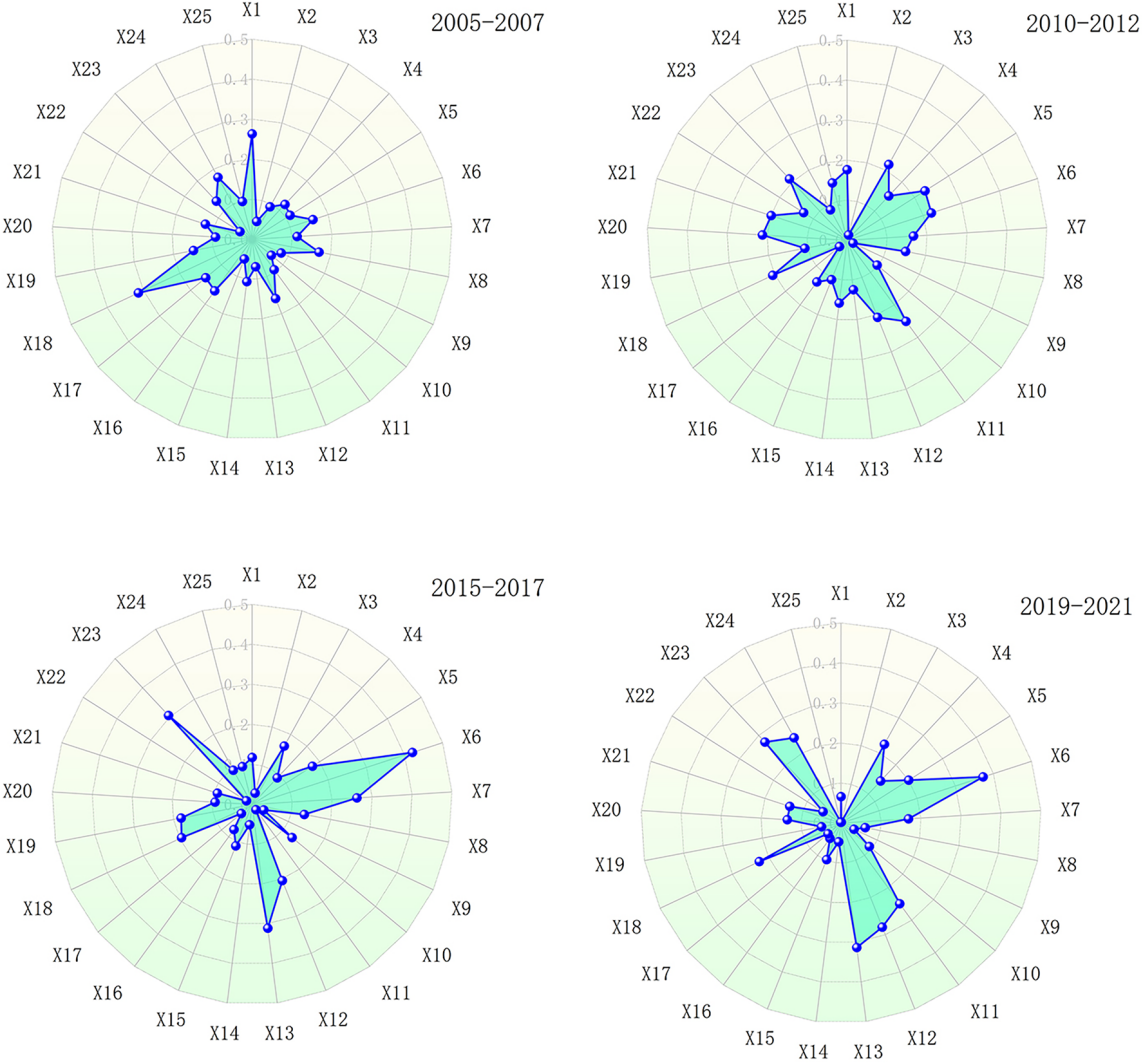
Q-values of WRCC driving factors in China from 2005 to 2021.

Geodetectors were employed to conduct a thorough analysis of 25 WRCC-related indicators in Chinese provinces over different time periods, as depicted in Fig. 4. The Q-statistic for each indicator reflects the trend of spatial variation, where a higher value indicates a more significant spatial heterogeneity of the corresponding indicator. Examining the trends in Q-statistic values, several indicators exhibit pronounced spatial heterogeneity variations. For instance, precipitation (X2), surface water supply (X3), water development and utilization rate (X5), per capita GDP (X6), urbanization rate (X7), among others, display substantial Q-statistic values across different time periods, indicating notable spatial heterogeneity and their significant impact on China's WRCC. Some indicators show fluctuations in Q-statistic values over different time periods. For instance, per capita GDP (X6) and sewage treatment rate (X25) exhibit significantly lower Q-statistic values in the 2019-2021 period, reflecting a decrease in their impact on China”s WRCC during this time frame. Per capita domestic water consumption (X10) and agricultural irrigation area (X12) demonstrate fluctuations across various periods, primarily influenced by different regional water resource management and agricultural development strategies.

Table 6 presents the driving effects of various criterion layers on the spatial differentiation of WRCC in China. Overall, the social and ecological sub-indicators exert a greater impact on the spatial differentiation of WRCC in China compared to the water resources and economic sub-indicators. Specifically, from 2005 to 2015, the ecological sub-indicator was a key limiting factor for WRCC in China, while since 2015, the social sub-indicator has become the primary limiting factor. The changes in factors constraining China's Water Resources Carrying Capacity (WRCC) are closely related to China's level of environmental concern. Specifically, China launched the ambitious Air Pollution Action Plan in 2013 and enacted new environmental protection laws in 2014, imposing strict controls on pollution activities. Considering the lag in policy implementation, environmental measures began to yield results gradually from 2015 onwards, which aligns with the findings of this study. Combining this information with Fig. 4 reveals that the main factors influencing the spatial differentiation of WRCC in China since 2005 include ecological water consumption (X18), water supply coverage rate (X11), per capita GDP (X6), economic density (X13), and industrial wastewater treatment capacity (X23). Examining the most crucial indicators influencing WRCC in China for each year shows that since 2005, the primary factors affecting WRCC in China follow the direction of ecological water consumption (X18), water supply coverage rate (X11), per capita GDP (X6). This reflects a shift in the essence of China's economic development from a focus on the economy to an emphasis on ecological protection, social development, and comprehensive progress in ecological civilization.

**Table 6:** Driving effects of criterion layers on China’s WRCC.

Criterion layers	Driving factor index			
	2005–2007	2010–2012	2015–2017	2019–2021
Water resources	0.634	0.789	0.583	0.638
Social	0.838	1.107	1.198	1.260
Economic	0.538	0.541	0.586	0.548
Ecological	1.119	1.298	1.078	1.121

## Discussion

### Identification of factors influencing inter-provincial WRCC in China

**Figure 5:**
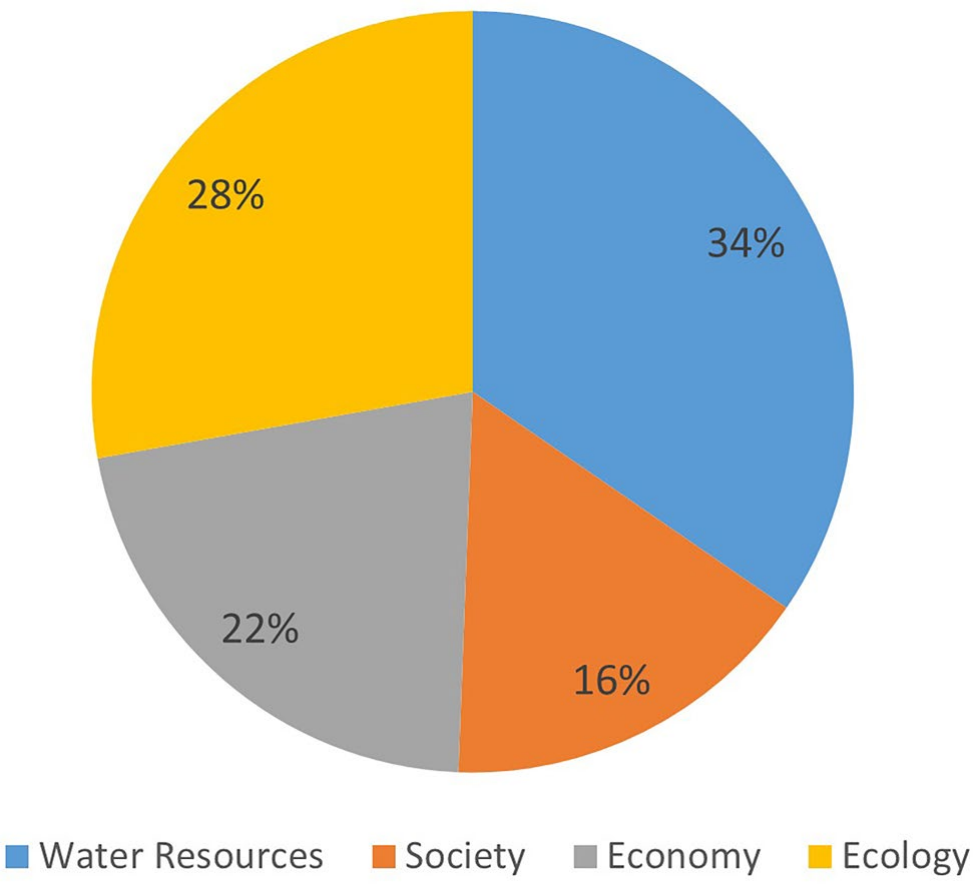
Comparison of criterion layer factor weights.

In the TOPSIS model, the influence of the research object can be represented by indicator weights. Figure 5 illustrates the indicator weights of the water resources, society, economy, and ecology sub-systems in the WRCC evaluation system of this study. A larger weight indicates a greater impact on China’s WRCC. It can be observed that the water resources sub-system has the greatest impact on China’s WRCC, followed by the ecology sub-system and the economy sub-system, while the influence of the society sub-system is relatively weak. This difference aligns well with real-world logic, although the weights of the four sub-systems are also subject to dynamic changes.

**Figure 6:**
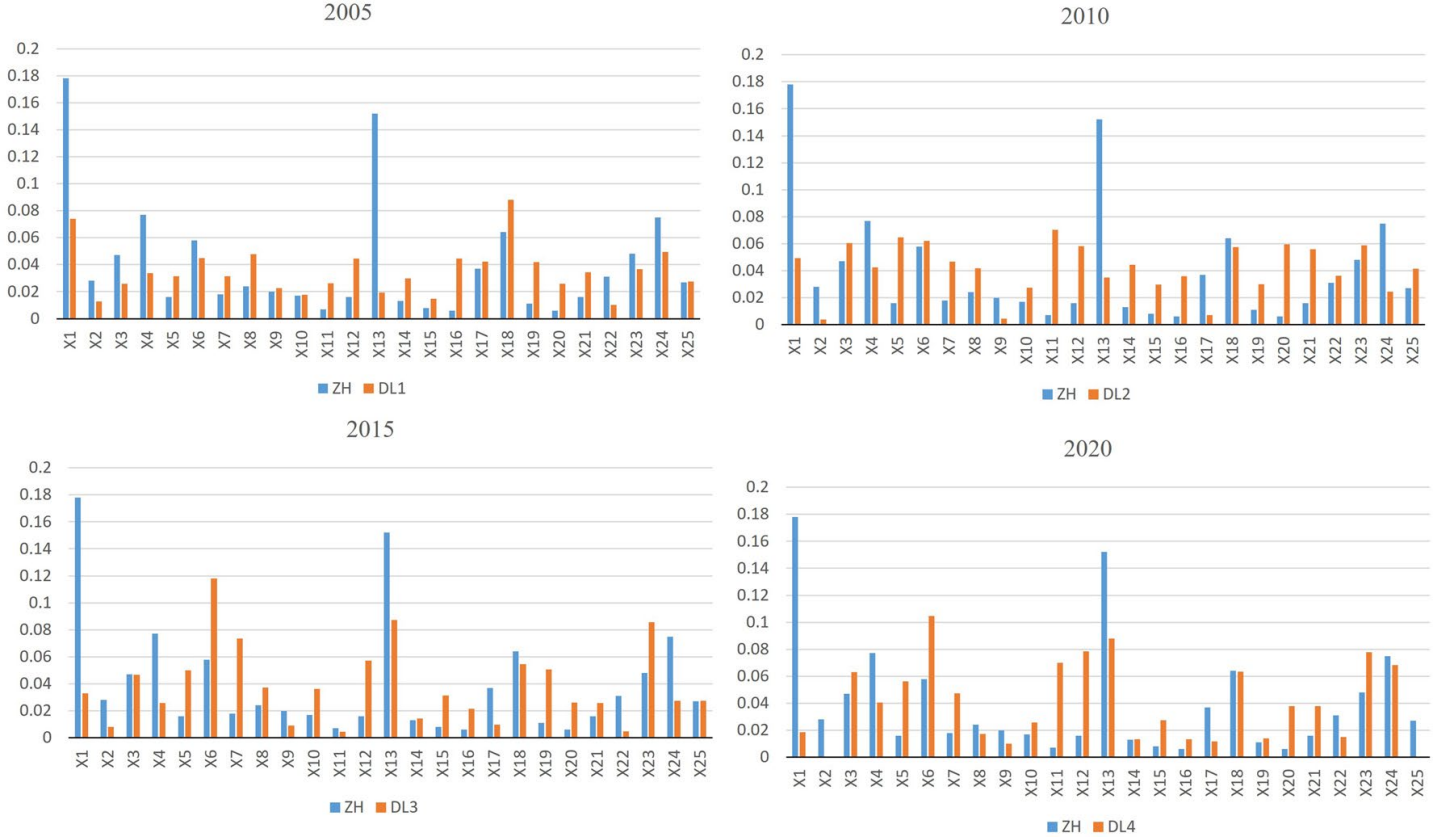
Comparison between indicator weight (ZH) and geographic detector factors (DL).

Apart from the weights of each indicator, the geographic detector also calculates the impact factors of each indicator, which can similarly represent the degree of influence of each indicator on China’s WRCC. As shown in Fig. 4, this study calculated the impact effects of various indicators for four time periods: 2005, 2010, 2015, and 2020. The values for each time period were compared with the indicator weights, and the results are presented in Fig. 6. It can be observed that, overall, the indicator weights are largely consistent with the geographic detector factors. Per capita water resources (X1), ecological water consumption (X18), per capita GDP (X6), economic density (X13), and industrial wastewater treatment capacity (X23) are identified as the core factors influencing WRCC. Additionally, it is noted that the geographic detector factors are subject to change, closely related to China’s environmental protection policies and economic transformation. Compared to the indicator weights, using the geographic detector to study the impact of various indicators on WRCC can better reflect the actual situation.

### Coupling degree of inter-provincial WRCC subsystems in China

Regional disparities in WRCC in China are significant. Coastal provinces in the eastern part of the country have maintained relatively high levels of WRCC due to their advantageous geographical location, developed economy, and advanced governance experience. In contrast, provinces in the western part of China have relatively limited WRCC due to harsh natural environments and lower economic levels. The coordination degree of water resources, economy, society, and ecology in each province of China also reveals this regional disparity. Coastal regions in the east demonstrate relatively high overall coordination in water, economy, society, and ecology, whereas coordination in western and northern regions is relatively low, with generally lower ecological coordination. This reflects regional disparities in water resources, heterogeneous economic structures, uneven levels of social development, and the collaborative challenges between ecological environments and socio-economic development. Areas with high population density generally exhibit lower coordination, highlighting the critical role of population factors in overall coordination. Additionally, it is observed that diversity in economic structures contributes to enhancing adaptability and resilience in coordination efforts.

### Comparative analysis of research results

Overall, during the study period, China’s water resource carrying capacity, especially in economically developed provinces along the eastern coast such as Beijing, Jiangsu, Zhejiang, and Guangdong, has significantly increased, with WRCC reaching level III or above in 2021. To assess the robustness of the results obtained in this study, various methods, as referenced by Lv, B. (2023) and others^47,48^, were employed to reevaluate WRCC for each province. The results, as shown in Table 7, indicate that although there are slight differences in WRCC indices among different research methods, the grading remains consistent, thus ensuring the reliability of the findings.

**Table 7:** Inter-provincial WRCC results using different research methods in China.

Province	Composite weight		Coefficient of variation		AHP method	
	Index	Grading	Index	Grading	Index	Grading
Beijing	0.401	IV	0.378	IV	0.403	IV
Tianjin	0.327	IV	0.309	IV	0.328	IV
Hebei	0.351	IV	0.332	IV	0.351	IV
Shanxi	0.270	III	0.252	III	0.271	III
Inner Mongolia	0.370	IV	0.352	IV	0.370	IV
Liaoning	0.304	III	0.285	III	0.305	III
Jilin	0.288	III	0.270	III	0.288	III
Heilongjiang	0.304	III	0.279	III	0.304	III
Shanghai	0.365	IV	0.342	IV	0.367	IV
Jiangsu	0.468	IV	0.439	IV	0.468	IV
Zhejiang	0.330	IV	0.310	IV	0.331	IV
Anhui	0.296	III	0.277	III	0.297	III
Fujian	0.332	IV	0.314	IV	0.333	IV
Jiangxi	0.296	III	0.283	III	0.297	III
Shandong	0.337	IV	0.314	IV	0.338	IV
Henan	0.353	IV	0.330	IV	0.354	IV
Hubei	0.331	IV	0.315	IV	0.331	IV
Hunan	0.309	III	0.292	III	0.309	III
Guangdong	0.353	IV	0.335	IV	0.354	IV
Guangxi	0.291	III	0.281	III	0.292	III
Hainan	0.268	III	0.256	III	0.269	III
Chongqing	0.279	III	0.261	III	0.281	III
Sichuan	0.286	III	0.267	III	0.287	III
Guizhou	0.262	III	0.248	III	0.263	III
Yunnan	0.275	III	0.258	III	0.275	III
Tibet	0.323	IV	0.319	IV	0.324	IV
Shaanxi	0.287	III	0.267	III	0.287	III
Gansu	0.266	III	0.252	III	0.267	III
Qinghai	0.245	II	0.231	II	0.246	II
Ningxia	0.236	II	0.228	II	0.237	II
Xinjiang	0.343	IV	0.318	IV	0.343	IV

## Conclusions and recommendations

### Key findings

Based on the TOPSIS model, this paper evaluates the WRCC index in China and analyzes the spatiotemporal evolution characteristics and driving factors of WRCC from 2005 to 2021 using coupling coordination and geographic detector methods. The conclusions are as follows:

- (1) During the study period, the number of provinces in China with WRCC at level IV increased from 4 to 14, while the number of regions at level I decreased from 18 to 0.
- (2) Since 2005, the WRCC index in China has continued to increase, mainly due to the transformation of the economic development mode in the study area and the emphasis on environmental protection policies. Especially in economically developed provinces along the eastern coast such as Beijing, Jiangsu, Zhejiang, and Guangdong, efforts have been made to promote industrial structure upgrading, continuously increase investment in water resource protection, and prioritize ecological civilization construction.
- (3) Criterion layer analysis indicates that the driving effect of social and ecological sub-indicators on the spatial differentiation of WRCC is significant, especially since 2015 when the social sub-indicator became the dominant factor. This reflects the evolution of China’s economic development from emphasizing economy to focusing on ecological and social development.
- (4) Regional disparities in WRCC in China are significant, with coastal provinces in the east maintaining relatively high levels of WRCC due to their geographical advantages, developed economy, and advanced governance experience, while provinces in the west have relatively limited WRCC due to harsh natural environments and lower economic levels.

### Recommendations

To promote the coordinated and sustainable development of China’s WRCC, we propose the following recommendations:

- (1) Formulate differentiated water resource governance policies. Considering the regional differences in WRCC among various provinces in China, it is recommended to develop differentiated water resource management policies. These policies should fully take into account the variations in natural conditions and local policies to enhance coordination and carrying capacity.
- (2) Improve water resource utilization efficiency. Given the relative lag in water resource sub-indicators, there is a suggestion to focus on improving water resource utilization efficiency in water resource management. Promoting technological innovation and adopting advanced water resource management techniques can reduce water pollution and enhance the sustainable utilization potential of water resources.
- (3) Enhance cross-regional coordination in water resource management. It is advisable to strengthen mechanisms for cross-regional coordination in water resource management, promoting more rational geographic distribution and more efficient economic allocation of water resources. Establishing joint prevention and control mechanisms can facilitate resource sharing among different provinces and coordinate actions in response to water resource emergencies, thereby reducing disparities in WRCC.
- (4) Encourage diversified industrial development. To enhance adaptability and resilience, the recommendation is to encourage provinces to develop diversified economic structures. Supporting and guiding diversification in industries can reduce excessive reliance on water resources, promote the optimization and upgrading of industrial structures, and achieve more synergistic development between water resources and the economy.

## Data Availability

Data will be available from the corresponding author upon reasonable request.
